# Evaluation of the Efficiency and Effectiveness of Three Minimally Invasive Methods of Caries Removal: An *in vitro *Study

**DOI:** 10.5005/jp-journals-10005-1226

**Published:** 2014-04-26

**Authors:** Ankush Ramnarayan Boob, M Manjula, E Rajendra Reddy, N Srilaxmi, Tabitha Rani

**Affiliations:** Senior Lecturer, Department of Pediatric Dentistry, CSMSS Dental College Aurangabad, Maharashtra, India; Professor, Department of Pediatric and Preventive Dentistry, Kamineni Institute of Dental Sciences (KIDS), Nalgonda, Andhra Pradesh, India; Professor and Head, Department of Pediatric and Preventive Dentistry, Kamineni Institute of Dental Sciences (KIDS), Nalgonda, Andhra Pradesh, India; Professor, Department of Pediatric and Preventive Dentistry, Kamineni Institute of Dental Sciences (KIDS), Nalgonda, Andhra Pradesh, India; Reader, Department of Pediatric and Preventive Dentistry, Kamineni Institute of Dental Sciences (KIDS), Nalgonda, Andhra Pradesh, India

**Keywords:** Carisolv, Papacarie, Knoop hardness number, Chemomechanical caries removal

## Abstract

**Background: **Many chemomechanical caries removal (CMCR) agents have been introduced and marketed since 1970s, with each new one being better and effective than the previously introduced. Papacarie and Carisolv are new systems in the field of CMCR techniques. These are reportedly minimally invasive methods of removing carious dentin while preserving sound dentin.

**Aim: **To compare the Efficiency (time taken for caries removal) and effectiveness (Knoop hardness number of the remaining dentin) of caries removal by three minimally invasive methods, i.e. hand excavation and chemomechanical caries removal using Carisolv and Papacarie.

**Materials and methods: **Thirty recently extracted human permanent molars with occlusal carious lesions were divided randomly in three equal groups and bisected through the middle of the lesion mesiodistally and excavated by two methods on each tooth.

**Results: **Statistically significant difference was present among three methods with respect to time and knoop hardness values (KHN) of the remaining dentin.

**Conclusion: **The Efficiency of Hand method is better compared to CMCR techniques and effectiveness of CMCR techniques is better than Hand method in terms of dentin preservation so the chances of maintaining vitality of the pulp will be enhanced.

**How to cite this article: **Boob AR, Manjula M, Reddy ER, Srilaxmi N, Rani T. Evaluation of the Efficiency and Effectiveness of Three Minimally Invasive Methods of Caries Removal: An *in vitro *Study. Int J Clin Pediatr Dent 2014;7(1):11-18.

## INTRODUCTION

Dental caries is one of the most common infectious diseases of mankind. Caries is a bioflm (plaque) induced acid dem-ineralization of enamel or dentin, mediated by saliva.^1^ It has consequences upon oral and general health of individuals (pain, impairment of function, reduced quality of life).^2^ Over the past 100 years, dentistry has matured from the original tenets of GV Black by moving from ‘extension for pre­vention’ to a ‘minimal intervention’ approach.^3,4^ The concept of conservative healthy tooth cavity preparation has gained popularity with the advent of adhesive resin bonding systems in which the retention and resistance form for cavity prepara­tion has also been minimized. Drilling often removes part of healthy tooth structure in addition to the decayed areas.^3,4^

There are various methods of caries removal which can be broadly divided into four major categories,^5^ i.e.

1. Mechanical, rotary
2. Mechanical, nonrotary
3. Chemomechanical
4. Photo-ablation

Chemomechanical caries removal (CMCR) is a non-invasive technique which eliminates infected tissues, pre­serving healthy dental structures, avoiding pulp irritation and patient discomfort.^5^ Many CMCR agents have been introduced and marketed since 1970s.^6-11^

Carisolv is a two-component gel designed to soften the altered dentin and make possible its gentle removal with specially designed scraping instruments.^12-15^

**Fig. 1:**
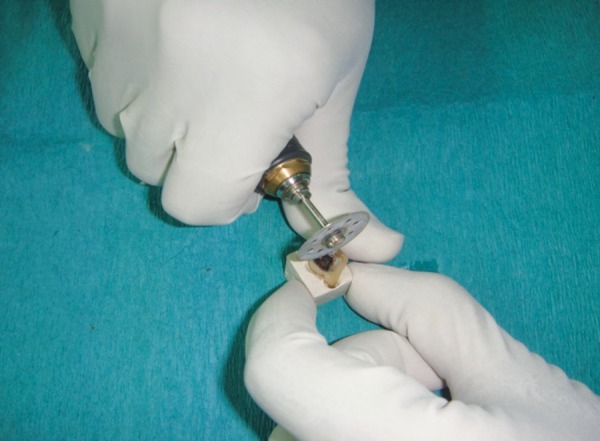
Sectioning of tooth

In 2003, a new CMCR agent called Papacarie (Formula Acão, São Paulo, Brazil) has been marketed. The product is a gel based papain, a proteolytic cisteine enzyme which presents antibacterial and anti-infammatory properties. Papain acts as a debris-removing agent, with no harmful effect on sound tissues because of the enzyme's specificity. It acts only on affected tissues, which lack the α1-antitrypsin plasmatic antiprotease that inhibits proteolysis in healthy tissues. The advantages include easy application and no specific instruments.^16-19^ This study was taken to evaluate and compare the Efficiency and effectiveness of the three minimally invasive caries removal methods.

## MATERIALS AND METHODS

The study was carried out in the Department of Pedodontics and Preventive Dentistry, Kamineni Institute of Dental Sciences Nalgonda, Andhra Pradesh, India, after written consent obtained from the local ethical committee and in Defence Metallurgical Research Laboratory (DMRL), Kanchanbagh, Hyderabad, Andhra Pradesh, India.

**Fig. 2:**
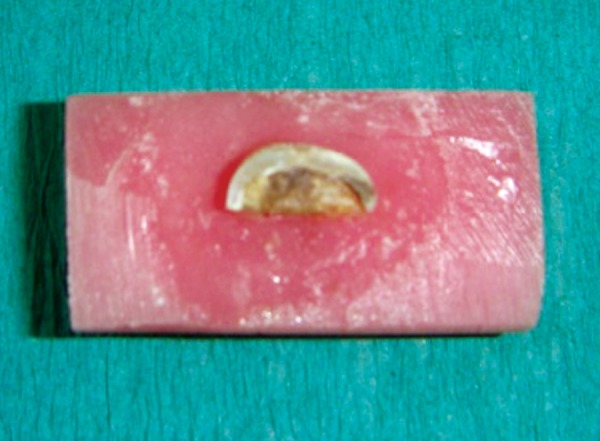
Stabilization of tooth occlusal view

**Fig. 3:**
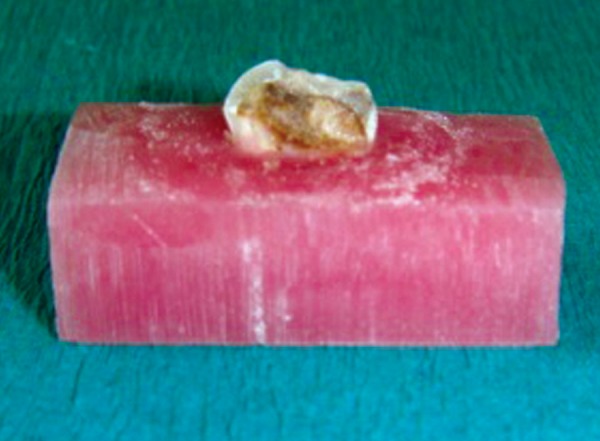
Stabilization of tooth side view

The independent variable investigated in this experiment was the method of carious dentin removal. The response or dependent variables were the time required to remove dentin measured in seconds (Efficiency analysis) and the knoop hardness number of the remaining dentin measured by a microhardness tester (effectiveness analysis). The experi­mental units were 31 recently extracted human permanent molars with occlusal carious lesions, bisected through the middle of the lesion mesiodistally and arranged in a split-tooth design (Figs 1 to 3). The teeth were stored for no longer than 30 days in phosphate buffered saline (pH = 7.2) to avoid the bias in the hardness of the carious lesions.

### Materials

**Fig. 4:**
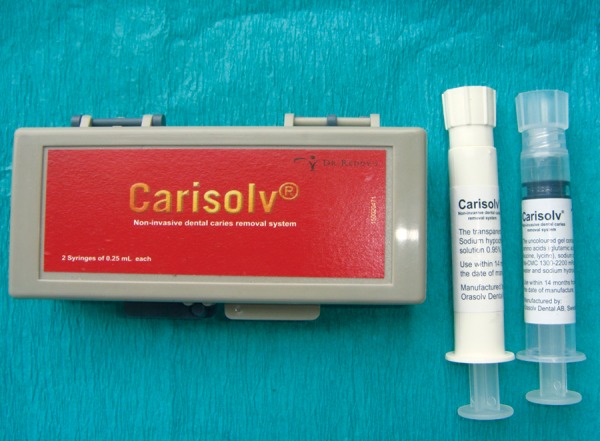
Carisolv system of caries removal

**Fig. 5:**
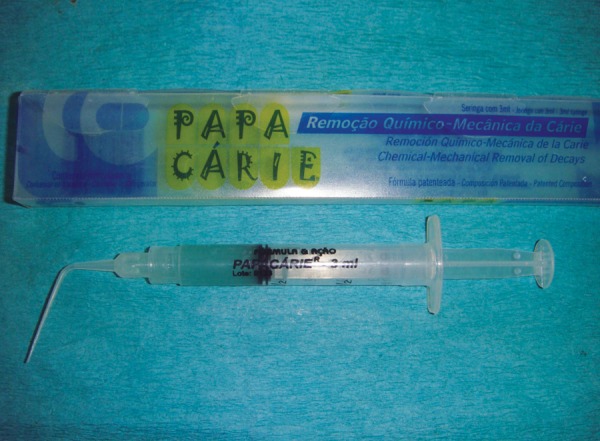
Papacarie system of caries removal

- Carisolv gel uncolored multimix (multimix gel-based chemomechanical caries removal system, MedIteam Dental AB, Sweden) (Fig. 4).
- Papacarie (Formula and Ação, Brazil) (Fig. 5).

### Caries Removal Procedure

The teeth were mounted in dental plaster blocks (plaster of paris; dental stone type II) with the roots of the teeth inside the plaster blocks and crowns completely outside. Teeth were then sectioned mesiodistally in the center of the carious lesion using diamond disks mounted in a straight handpiece into two equal-sized carious lesions. After sectioning the tooth mesiodistally, the depth of each carious half was measured with a divider and scale. To further reduce the bias, the lesions which were not of approximately same depth were discarded. So, the final sample size was 60 halves. The two halves of the each carious lesion were embedded in two separate cold cure acrylic blocks such that the acrylic resin should be around the tooth structure except on the carious lesion (*see *Figs 1 to 3). One half of the each tooth was randomly excavated by two different methods of caries removal, i.e. Conventional, Carisolv or Papacarie and the operator, was blind to the technique used. Sixty samples were divided into three groups as group 1, 2 and 3 with each group having 20 samples.

**Figs 6A and B:**
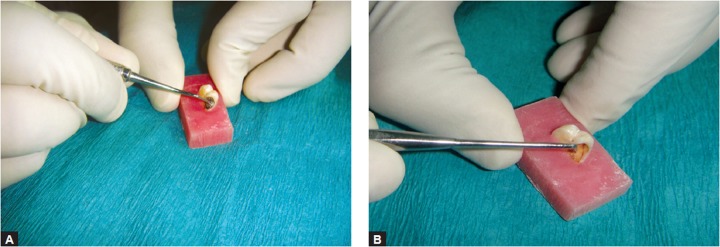
Caries excavation by the hand excavation method with spoon excavator

Each group was subdivided into two as sub-group A, subgroup B in group 1, subgroup C, subgroup D in group 2, subgroup E, subgroup F in group 3.

- *Group 1*: Comparison of conventional (subgroup A) versus Carisolv (subgroup B) method.
- *Group 2*: Comparison of conventional (subgroup C) versus Papacarie (subgroup D) method.
- *Group 3*: Comparison of Papacarie (subgroup E) versus Carisolv (subgroup F) method.

After caries excavation in all methods, toileting of the cavity was carried out with sterile cotton pellets.

## CARIES EXCAVATION

### Hand Method

After the sample was embedded in the acrylic resin block, the carious dentin excavation was started in one-half randomly with the help of spoon excavator (Hu-Friedy) and accomplished in circular scratching movements from the dentinoenamel junction to the cavity foor using spoon excavator depending on the lesion size. Excavation was completed when dentin at the cavity floor was resistant to probing, following the criteria of hard texture (Figs 6A and B).

### Carisolv Method

**Fig. 7:**
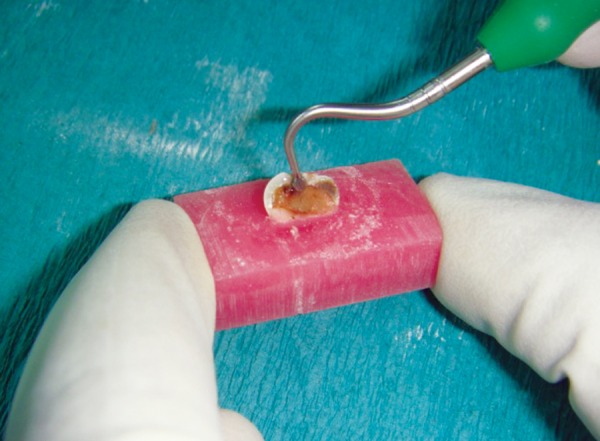
Caries excavation with Carisolv and specific instrument recommended by Mediteam

Caries excavation was done by Carisolv method accord­ing to manufacturer's instructions. The dentin caries was covered with Carisolv gel and, after 30 seconds, the carious dentin was gently scraped away with the specially designed hand instruments supplied by the manufactures [MediTeam (Sweden)] to remove softened carious tissue. The procedure was repeated until the gel became clear and the complete excavation of the caries is confirmed by tactile and visual method of caries detection (Fig. 7). A sharp explorer was used to confirm that the cavity was free of caries. Once the cavity was confirmed free of caries, the gel was removed with a cotton pellet soaked in water.

### Papacarie Method

**Figs 8A and B:**
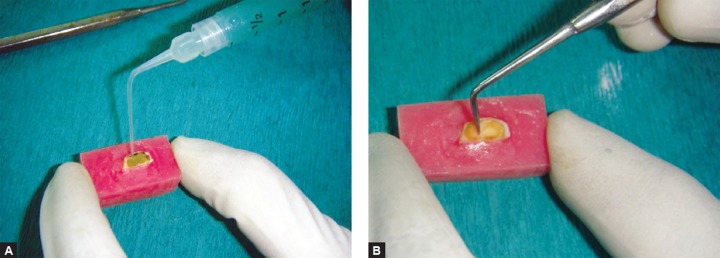
Caries excavation with Papacarie and spoon excavator

The carious cavity was first filled with Papacárie® gel (Figs 8A and B) and it was allowed to act for 40 to 60 seconds according to manufacturer's instructions. The softened decayed dentin was scraped away with the help of a sharp spoon excavator. The gel was reapplied as many times as necessary, i.e. until a light color was observed. The dark color indicates that the decomposition of the decayed tissue was still in process. When the gel reached an unchanged light color, the complete excavation of the caries was confirmed by visual and tactile method of caries detection. Once the cavity was confirmed free of caries, the gel was removed with a cotton pellet soaked in water.

## EFFICIENCY ANALYSIS

The time taken for both the procedures were measured from start of caries removal till the cavity was confirmed to be free of caries with the help of a stop watch and was recorded.

### Evaluation of the Cavity for Complete Caries Removal

A sharp explorer was used and moved across the dentinal wall. The caries removal wa s considered to be complete when the explorer does not stick to the dentin, and there was no ‘tug-back’ sensation.^20-24^ This method gives an idea about the nature of the carious lesion. Presence of soft texture suggests that an active carious lesion is present. The most reliable clinical method for the determination of the activity and extent of the carious lesion is by the tactile discrimi­nation of the surface texture after the lesion is exposed.^22,25,26^A discolored surface that is soft in nature and irregular on exploring is suggestive of an active carious lesion. Accord­ing to these guidelines, the discrimination of the carious lesion can be done very easily and is considered one of the most reliable and easily available methods for the detection of caries. A discolored surface that is hard and smooth to explorer touch is an arrested lesion and may not require any treatment. This method has an advantage that it does not need any additional equipment.^22,25,26^

## EFFECTIVENESS ANALYSIS

For microhardness testing, the samples with the teeth halves embedded in the acrylic resin were planed in water cooled mechanical grinder slightly above the maximum depth of the carious lesion and with the silicon carbide waterproof papers of 200, 320, 500, 600, 800, 1000 grits roughness (Jawan Brand and John Oakey and Mohan Ltd, India). The samples were then polished with Al_2_O_3_ (Mager Alumina Alpha AP-358, Mager Scientific Inc., Dexter, MI) and the polishing cloths (4 × 50; 4 × 100).

The Knoop hardness number (KHN) was obtained for all the samples in a microhardness tester (MMT3, Matsuzawa Co. Ltd; Model No: MMT3, MM0708, Japan) with a 50 gm static-load applied for 20 seconds by independent operator who was blind to the different group as well as subgroups. Three indentations were made from the deepest point of the cavity foor to the pulp chamber roof, one in the region of the remaining dentin after caries excavation, one in the adjacent dentin and one on the healthy dentin.^27^ The average of the three readings was taken as the mean KHN of the remain­ing dentin. The KHN values are derived using the equation KHN = 14229.K/L^2^, where K is the applied load in grams, and L is the indentation length, in micrometers.^28^ The KHN values in this study were obtained directly from the digital readings on the screen of the tester.

Data were analyzed for the Efficiency and effectiveness using Windows Statistical Package for Social Sciences (SPSS) Version 16.0. Independent t-test, one-way ANOVA test and Newman-Keuls multiple posthoc procedures were used.

## RESULTS

### Efficiency

When total time taken for all 60 samples was taken into consideration, the mean time taken for all teeth was 543.42 ± 161.76 seconds while the hand excavation and Carisolv were the minimum and maximum time consuming procedures respectively.

**Graph 1:**
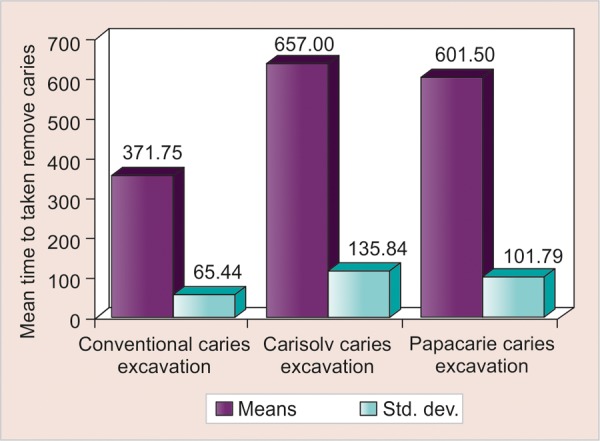
Comparison of three methods with respect to time taken to remove caries

**Graph 2:**
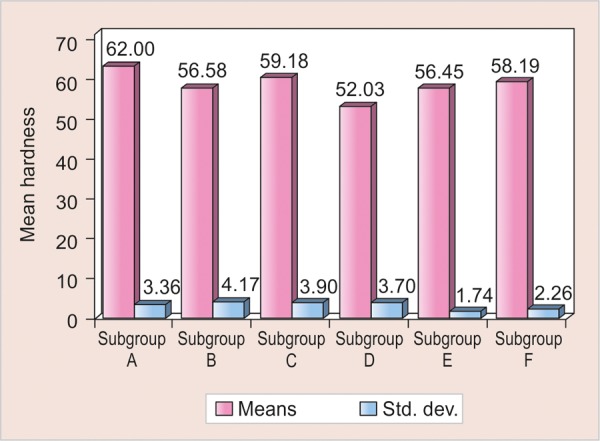
Overall comparisons of all the subgroups with respect to KHN

**Graph 3:**
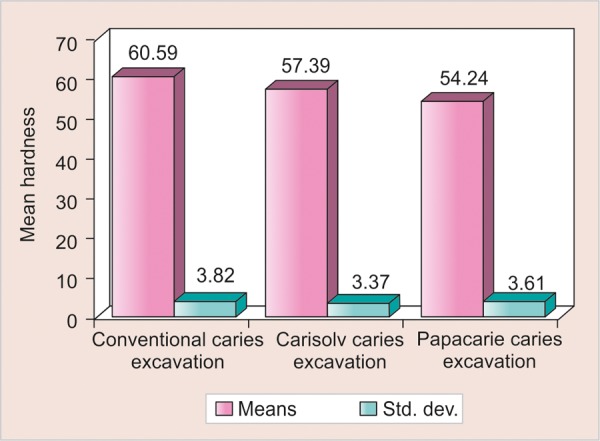
Comparison of three methods with respect to hardness values

Comparison of Hand, Carisolv and Papacarie methods with respect to time was done by one-way ANOVA test which showed statistically significant difference among the groups (Graph 1). Newman-Keuls multiple posthoc proce­dures were performed which confirmed that the significant difference was present between Hand method of caries excavation and Carisolv method, and also between Hand method of caries excavation and Papacarie method.

### Effectiveness

The mean KHN of the remaining dentin for all 60 samples was 57.40 ± 4.40 (Graph 2).

Comparison of the three methods with respect to KHN of the remaining dentin was done by one-way ANOVA test (Graph 3) which was confirmed by Newman-Keuls multiple hoc procedures.

## DISCUSSION

During the invasive treatment of caries using high speed instruments, the dental surgeon is forced to destroy the sound tooth structure. The concept of minimally invasive dentistry is based on removing caries with method that minimize the loss of sound enamel and dentin.^29^ Carious dentin is composed of an outer infected layer that is irreversibly denatured and an inner affected layer that is capable of remineralization.^30^Preserving affected carious dentin during cavity preparation procedure is useful because of its lower permeability and sclerotic nature compared with healthy dentin. It protects the pulp from any remaining bacteria in the affected dentin. With the advent of newer adhesive restorative materials minimal removal of the sound tissue may increase the performance of the tooth-restoration complex by retaining the maximum amount of healthy tissue.^30,31^ This led to increasing interest in alternative methods of caries removal and an evolution of chemomechanical method of caries removal.

Because natural lesions were used, it was not possible to standardize all variables of the sample, e.g shape and activity status of the lesions. Therefore, split-tooth methodology was used to minimize these variables as the source of carious dentin, thus allowing for comparisons to be made between different, paired excavation methods.^8,13,23,28^. In this study, only occlusal carious lesions extending till the middle third of the dentin were selected and split tooth methodology was followed, i.e. carious lesion on each tooth was split mesiodistally in to two equal halves so that the depth and extent of the lesion could be standardized to the maximum extent.^28^ After sectioning the tooth mesiodistally, the depth of each carious half was measured with a divider and scale. To further reduce the bias, the lesions which were not of approximately same depth were discarded.^28^

After sectioning, it was ascertained that none of the carious lesions were in close proximity to the pulp and sufficient thickness of remaining dentin was left so as to provide enough thickness to check the KHN number of the remaining dentin. Knoop and Vickers microhardness is suitable for determining the hardness of very brittle materials, such as dental hard tissue.^27,32,33^ The hand excavation was done with a standardized Hu-Friedy spoon excavator and Carisolv assisted excavation was done with instrument specially designed for Carisolv excavation. The instrument for Carisolv allowed for scraping action in two or more directions in contrast to regular excavators, which only have one working direction. These instruments have a 90° edge, and a blunt cutting Profile, which reduces the risk of removing intact dentin, as compared to treatment with conventional excavators and drills.^23,24^ For caries removal by the Papacarie method, Hu-Friedy spoon excavators were used as specific set of instruments is not required according to manufacturer.

The evaluation of the complete removal of caries was done by careful examination for the presence of caries using visual examination and a sharp probe. For caries removal to be judged as complete, the probe should not catch any remaining carious dentin.^25,26^ The results of our study showed that the Carisolv and Papacarie-assisted excavation taken longer time for caries removal than the conventional Hand excavation method. This longer time was justified as the manufacturer did not specify the minimum appli­cation time, but rather stated that the cavities should be treated until the gel becomes clear, to be considered caries free.^15-17,28,32,33^ Multiple applications of the Carisolv and Papacarie gel for complete caries removal may also be a reason for the increased time needed. However, it was neces­sary because the gel becomes blurred during the procedure and inspection of the cavity is difficult without rinsing it off. In addition, the dull appearance of the dentin cavity walls left after each Carisolv as well as Papacarie rinse-off may cause difficulties in caries removal evaluation. The dull appearance may be due to a thinner smear layer left on the Carisolv and Papacarie treated dentin as well as the rougher surface produced.^17,18,23,28,32,34-37^

Microhardness analysis has been used as a method to assess loss and reincorporation of minerals to the dental tissue, because the reduction in the numerical hardness value presents a linear relation to mineral loss.^27,32,33^ Obtaining a KH measurement of the cavity surface was impossible, recordings were obtained below the cavity foor; the hardness of the subsurface at a point 25 μm below the cavity foor was used as that of the cavity foor.^27,32^ Knoop Hardness Number was measured at three points in each treated cavity by application of 50 gm load for 20 seconds by means of hardness tester by an independent observer. The mean of the measurements was used as the KHN of the dentin. In our study, also it was found that Carisolv and Papacarie excavation left more amount of demineralized dentin as compared to hand excavation. This may be attributed to the chemical nature of Carisolv and Papacarie which was also confirmed by the another parameter of the study in which hardness of the remaining dentin was checked using KH tester and it was found that the hardness number was significantly lower for the Carisolv and Papacarie excavated group, suggesting that it is less mineralized than the underlying sound dentin. On intercomparison of these two materials between the Carisolv and Papacarie, it was found that Papacarie left more demineralized dentin but statistically significant difference was not present. It was confirmed by the KHN of the samples. Thus, we can say that conventional hand excavation leaves much harder dentin as compared to Carisolv as well as Papacarie excavation.

Splieth et al^38^ and Cedurlend et al^39^ verified caries removal according to color and hardness of the lesion with a sharp explorer. The hardness of dentin was checked until rather a hard texture was reached or a sharp scratching sound was heard.

## CONCLUSION

Based on the findings of the study, following conclusions were drawn:

- The conventional Hand excavation method takes less time for caries removal than the chemomechanical caries removal methods, i.e. Carisolv and Papacarie, which is statistically significant.
- KHN of Hand excavation method was more as compared to Carisolv and Papacarie which signifies that less amount of demineralized dentin is present after caries removal as compared to Carisolv and Papacarie excavation methods.
- Efficiency as well as effectiveness of Papacarie was better than Carisolv.
